# The Prognostic Value of Plasma Galectin-3 in Chronic Heart Failure Patients Is Maintained when Treated with Mineralocorticoid Receptor Antagonists

**DOI:** 10.1371/journal.pone.0119160

**Published:** 2015-03-18

**Authors:** François Koukoui, Franck Desmoulin, Michel Galinier, Manon Barutaut, Celine Caubère, Maria Francesca Evaristi, Gurbuz Murat, Rudolf De Boer, Matthieu Berry, Fatima Smih, Philippe Rouet

**Affiliations:** 1 INSERM I2MC, UMR 1048, Université UPS, Equipe, Obésité et insuffisance cardiaque: approches moléculaires et cliniques, F-31432 Toulouse, France; 2 CHU de Rangueil, Service de Cardiologie A, F-31432 Toulouse, France; 3 Department of Cardiology, University Medical Center Groningen, University of Groningen, Groningen, The Netherlands; University of Glasgow, UNITED KINGDOM

## Abstract

**Objective:**

Galectin-3 (Gal-3) is considered as a myocardial fibrosis biomarker with prognostic value in heart failure (HF). Since aldosterone is a neurohormone with established fibrotic properties, we aimed to investigate if mineralocorticoid receptor antagonists (MRAs) would modulate the prognostic value of Gal-3.

**Methods:**

The IBLOMAVED cohort comprised 427 eligible chronic HF patients (CHF) with echocardiography and heart failure biomarkers assessments (BNP). After propensity score matching CHF patients for cardiovascular risk factors, to form balanced groups, Gal-3 levels were measured at baseline in plasma from patients treated with MRAs (MRA-Plus, n=101) or not (MRA-Neg, n=101). The primary end point was all-cause mortality with a follow-up of 3 years.

**Results:**

Gal-3 in plasma from these patients were similar with median values of 14.0 ng/mL [IQR, 9.9–19.3] and 14.4 ng/mL [IQR, 12.3–19.8] (P = 0.132) in MRA-Neg and MRA-Plus, respectively. Patients with Gal-3 ≤17.8 ng/mL had an HR of 1 (reference group) and 1.5 [0.4–5.7] in MRA-Neg and MRA-Plus, respectively (p=0.509). Patients with Gal-3 ≥ 17.8 ng/mL had an HR of 7.4 [2.2–24.6] and 9.0 [2.9–27.8] in MRA-Plus and MRA-Neg, respectively (p=0.539) and a median survival time of 2.4 years [95%CI,1.8–2.4]. Multivariate Cox proportional hazard analysis confirmed that MRA and the interaction term between MRA treatment and Gal-3 >17.8 ng/mL were not factors associated with survival.

**Conclusions:**

MRA treatment did not impair the prognostic value of Gal-3 assessed with a 17.8 ng/mL cut off. Gal-3 levels maintained its strong prognostic value in CHF also in patients treated with MRAs. The significance of the observed lack of an interaction between Gal-3 and treatment effect of MRAs remains to be elucidated.

## Introduction

Galectin-3 (Gal-3), a member of the family of beta-galactoside-binding lectins, is a 30 kDa glycoprotein with a carbohydrate recognition domain of 130 amino acids that plays a role in many biological processes, including fibrosis [1–3]. Gal-3 provides a link between inflammation and fibrosis. Macrophage-derived Gal-3 was first suggested to be an important mediator in cardiac fibrosis by inducing cardiac fibroblast proliferation and collagen deposition resulting in HF development and progression [4]. Gal-3 was proposed as a biomarker of heart fibrosis that could predict outcome of heart failure (HF) [5]. In several cohorts of acute HF [6, 7] and chronic HF [8], Gal-3 was shown to be a powerful predictor of mortality. In most studies, Gal-3 had independent prognostic value when corrected for common risk factors such as age, gender and (NT-pro)BNP. Further, elevated Gal-3 in subjects from the general population has been associated with increased mortality [9, 10] and new-onset HF [10]. Recently, Gal-3 was approved by the US Food and Drug Administration as a new biomarker for HF risk stratification and has received a Class IIb recommendation for additive risk stratification in AHA/ACC guidelines [11].

Gal-3 has established interaction with specific pathophysiology in the HF syndrome. For instance, a strong interaction with kidney function seems to exist [12]. Further, in HF patients, Gal-3 levels have been shown to be significantly correlated with serum markers of cardiac extracellular matrix turnover [13]. Experimental evidences clearly link Gal-3 to fibrosis in the heart [14], but also renal [15], liver [16], and lung fibrosis [17].

Aldosterone is a central player in fibrosis [18]. Gal-3 has been shown to mediate the aldosterone-induced fibrosis response [19]. Therefore, we aimed to evaluate if the prognostic value of Gal-3 in chronic heart failure patients, either treated or not treated by mineralocorticoid receptor antagonists (MRAs), would be different. MRAs are recommended in the ESC and AHA/ACC guidelines as an additional therapeutic option to improve outcomes in patients with HF and reduced ejection fraction [11, 20]. The anti-fibrotic action of MRAs has been proposed as one of the mechanisms linked to the clinical benefit of aldosterone blockade [21]. A subanalysis of the RALES study showed that high baseline serum levels of markers of matrix turnover were significantly associated with poor outcome, and these markers were amenable to spironolactone therapy [22]. Given the intimate relation between aldosterone, fibrosis, and Gal-3, and the differential effects of MRAs in patients with active fibrogenesis, we hypothesized that the predictive value of Gal-3 in HF patients may be influenced by the use of MRAs. A recent subanalysis from the HF-ACTION study, however, showed no differential response of MRAs in patients with Gal-3 below or above the FDA-cleared cutpoint of 17.8 ng/mL [23]. Because this study was limited to the pre-specified inclusion/exclusion criteria of the HF-ACTION study, to date, an interaction between effects of anti-aldosterone treatment and Gal-3 has not been definitely demonstrated in HF patients. Our objective was to investigate the effect of MRAs on the prognostic value of Gal-3 in a contemporary cohort of chronic HF patients routinely seen at a University Hospital in France.

## Patients and Methods

### Ethics statement

The IBLOMAVED study was registered in a clinical database (ClinicalTrials.gov NCT01024049) and conform to the ethical guidelines of the 1975 Declaration of Helsinki. The protocol was approved by the institution’s human research (COSSEC) and regional ethics committee (Comite de Protection des Personnes (CPP) # DC 2008–452). Written informed consent was obtained from all participants and/or their legally authorized representatives.

### Study design

This is a retrospective investigation of interaction between MRA treatment and the prognostic value of Gal-3 in a subset of CHF patients from the IBLOMAVED study [24]. The IBLOMAVED cohort comprised 686 patients admitted between July 2007 and May 2013 to the cardiology department at Rangueil Hospital, Toulouse, France. Inclusion criteria required absence of stroke within the last 6 months, acute liver failure within the last 6 months or known chronic hepatic failure (alanine aminotransferase or aspartate amino transferase 5 times the upper regular limits), history for alcohol abuse or drug addiction, cancer or a diagnosis of cancer within 5 years, history of recent or current drug or alcohol abuse, participation in any clinical trial within 30 days prior to admission, hematological pathology (myelodegenerative syndrome, severe anemia (Hb, 8 g / 100 mL) or severe neutropenia (neutrophile count, 1000 cells /mL), thrombocypenia (platelet count,7500 plts/mL). Baseline demographic data, admission vital signs, clinical history, assessment of cardiovascular risk factors and admission medication were recorded. Biochemical data including BNP levels were obtained at admission. Venous blood samples were obtained during the morning within 24 hours following admission. In this cohort, 427 patients had a medical history of HF at admission. This cohort comprised patients from all AHA/ACC HF classification groups to provide a wide spectrum of patients for HF biomarkers studies. All the 686 patients underwent transthoracic echocardiography for left ventricular ejection fraction (LVEF) assessment. We used a threshold value of LVEF< 45% to select stable systolic chronic heart failure (CHF) patients (n = 427). Because of the multivariate filtering (propensity score), patients with missing data were discarded (n = 74). Patients were sorted into two groups: treated by mineralocorticoid receptor antagonists (MRA-Plus; n = 127) or not treated with this drug (MRA-Neg; n = 230). Finally, 101 patients were selected in each group by the propensity score matching method.

### Primary end point

The primary end point was all-cause mortality during follow-up.

### Measurement of galectin-3

Galectin-3 (Gal-3) was measured at baseline using an automated immunoassay on the VIDAS analyzer (bioMérieux, Marcy l’Etoile, France). The VIDAS Galectin-3 kit quantitatively measures the concentration of human galectin-3 levels in EDTA plasma. This assay has a measuring range of 3.3 to 100 ng/mL, high repeatability (CV about 1%) and reproducibility (CV about 5%), without cross-reactivity with collagens or other members of the galectin family. Commonly used HF medication does not interfere with the assay.

### Statistical analysis

Analyses were performed using Statistical R (version 3.0.1; http://www.r-project.org) or Medcalc (version 12.7.7; Medcalc Software bvba, Ostend, Belgium). Continuous variables were expressed as means ± standard deviation for normally distributed data or as medians with 95% confidence interval. Propensity scores were generated from logistic regression analysis (MatchIt package version 2.4.21)[25] with MRA treatment as dependent variable and included age, gender, obesity, diabetes, arterial hypertension (AHT), dyslipidemia, ischemic cardiomyopathy (ICM), left ventricular ejection fraction (LVEF), heart rate (HR), blood creatinine concentration (Creat) as predictors. Pairs of MRA-Neg and MRA-Plus patients were formed with propensity scores differing by at most 0.25 of the standard deviation of the logit of the propensity score [26]. Non parametric Mann-Whitney rank sum test and Chi-square test were used for statistical testing of continuous and categorical variables, respectively. Survival curves were constructed using the Kaplan-Meier method and compared with the log-rank test. Patients were categorized based on a Gal-3 threshold value of 17.8 ng/mL [27]. Additional categorization was made based on MRA treatment and patients not using MRAs with Gal-3 ≤ 17.8 ng/mL constituted the reference group (HR = 1.0). Univariate Cox regression was applied to evaluate the hazard ratio of covariates adjusted for age and gender. Mutivariate Cox proportional hazards models 1 to 3 were performed with variables that obtained a p<0.2 as result of the univariate analysis. Stepwise regression was performed with the variable entered if its associated significance level was less than P < 0.1 and removed if its associated significance level was greater than P > 0.2 in the regression model.

## Results

In the CHF subset of the IBLOMAVED cohort, about one third of the patients were treated by MRAs (123/353). The propensity score matching method was used to balance demographic and clinical criteria between MRA-Neg and MRA-Plus patients (Table 1). After matching, the selected 101 pairs of MRA-Neg and MRA-Plus patients presented similar characteristics for the matched variables. Treatments with beta blockers, ACE inhibitors and loop diuretics (Furosemide) were more frequently used in MRA-Plus patients. BNP measured at admission were similar in both groups (Table 1). Gal-3 retrospectively measured in plasma of these groups of patients were similar with median values of 14.0 ng/mL [IQR, 9.9–19.3] and 14.4 ng/mL [IQR, 12.3–19.8] (P = 0.132) in MRA-Neg and MRA-Plus, respectively (Table 1).

**Table 1:** Baseline characteristics of CHF patients.

	MRA-Neg	MRA-Plus	
	(n = 101)	(n = 101)	P
Age, y ±SD	59 ± 15	57 ± 11	0.299
Gender, % female	23	20	0.731
Obesity, %	23	25	0.869
Diabetes, %	23	22	1.000
AHT, %	36	33	0.767
Dyslipidemia, %	41	35	0.468
ICM, %	41	41	0.886
LVEF, % [95% CI]	30 [28–34]	30 [26–31]	0.346
LVEF < 30, %	41	46	0.571
HR, bpm [95% CI]	70 [69–80]	72 [70–76]	0.554
NYHA 2	55	60	0.627
NYHA 3	32	32	0.879
NYHA 4	12	8	0.541
ICM, %	41	41	0.886
Acute pulmononary*	34	34	0.882
ARA II	13	13	0.834
Calcium blockers	7	7	0.782
AVK drugs	34	35	0.961
Antiplatelet Agents	39	46	0.325
Statines	49	44	0.572
Beta blockers	67	86	0.003
ACE inhibitors	60	80	0.003
Furosemide	60	90	<0.001
Creat, μM [95% CI]	95 [90–102]	97 [88–102]	0.910
Serum sodium, mM	139 [137–139]	137 [137–138]	0.005
SGOT, IU/l [95% CI]	28 [25–32]	27 [25–30]	0.757
BNP, pg/ml [95% CI]	399 [210–206]	364 [261–563]	0.724
Gal-3, ng/ml [95% CI]	14.0 [9.9–19.3]	14.4 [12.3–19.8]	0.132

MRA-neg, Patients not treated by mineralocorticoid receptor antagonist; MRA-plus, Patients treated by mineralocorticoid receptor antagonist; AHT, arterial hypertension; ICM, ischemic cardiomyopathy; Creat: creatinine; LVEF, left ventricular ejection fraction; BNP, brain natriuretic peptide; Gal-3, Galectin-3; HR: heart rate; NYHA, New York Heart Association classes; SGOT, serum glutamic oxaloacetic transaminase; ARA II, angiotensin II receptor antagonists; AVK drugs, Anti vitamin K drugs; ACE inhibitors, angiotensin-converting-enzyme inhibitors. Propensity score was used to match patients based on the following variables: age, gender, obesity, diabetes, arterial hypertension (AHT), dyslipidemia, ischemic cardiomyopathy (ICM), left ventricular ejection fraction (LVEF), blood creatinine concentration (Creat). *Acute pulmonary edema occurring during the last month preceding the inclusion.

**Fig 1:**
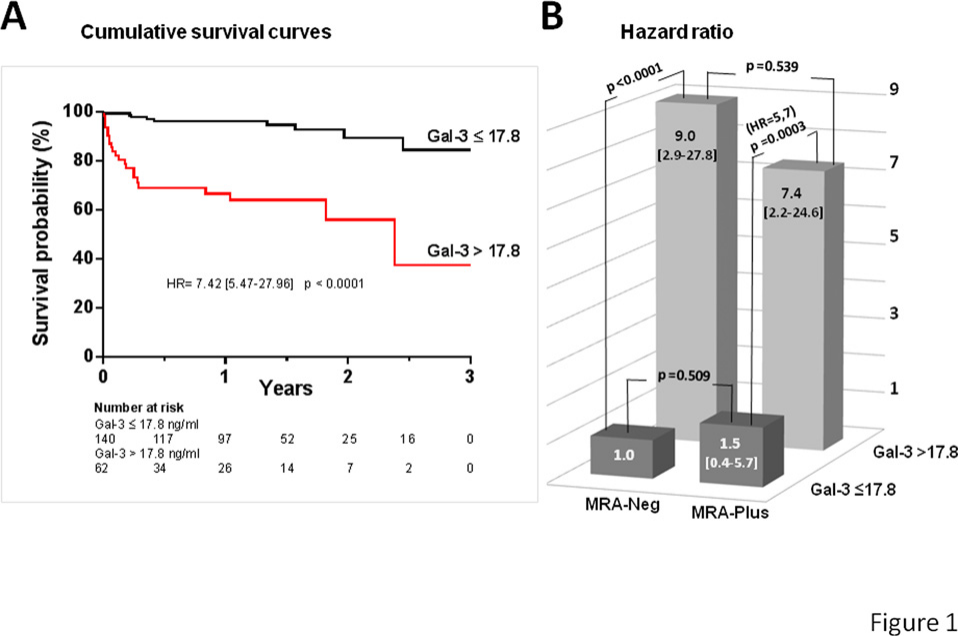
Survival analysis of cohort by the combination of MRA treatment and Gal-3 stratification. (A) Patients were categorized in two groups according to baseline concentration of Gal >17.8 ng/mL. Hazard ratio (HR) for patients with Gal-3 > 17.8 ng/mL was 7.42 [95%CI, 5.47–27.96]; p<0.0001. (B) Comparison of HR between the four groups of patients, according to the MRA treatment (MRA-Neg or MRA-Plus) and the Gal-3 level (Gal-3≤17.8 ng/mL or Gal-3>17.8 ng/mL). P values of differences between groups are indicated. The group of patients with Gal-3 ≤ 17.8 ng/mL and without MRA treatment constitutes the reference group (HR = 1).

Median follow-up was 1.13 years (14 months) [IQR, 0.46–1.65 (6–20 months)]. Out of the 202 patients, 31 (15%) were censored for primary endpoint and 171 (85%) were end-of-study censored. The concentration of Gal-3 was higher in patients who died than in survivors with median values of 29.6 ng/mL [IQR, 14.8–33.1] and 13.8 ng/mL [IQR, 10.5–17.6] for the former and later, respectively, P<0.001. Patients were stratified according to baseline Gal-3 concentration ≤ 17.8 ng /mL which corresponds to the cut off with the low risk of adverse outcome in HF patients [8]. Out of the 202 patients, 140 (69%) had a Gal-3 concentration ≤ 17.8 ng/mL. Survival analysis showed that patients with a Gal-3 >17.8 ng/mL presented a higher risk of death with a HR of 7.42 [95%CI, 5.47–27.96] and a median survival time of 2.4 years [95%CI,1.8–2.4] (Fig. 1A). Similar HR values were obtained with univariate Cox proportional hazard analysis adjusted for age and gender (Table 2) or with different multivariate models which included clinical, paraclinical and medication factors (Table 3). Clearly, MRA treatment was not associated with survival of patients (Table 2). Indeed, the prognostic value of Gal-3 was not significantly affected by MRA treatment (Fig. 1B). Multivariate Cox proportional hazard analysis confirmed that MRA and the interaction term between MRA treatment and Gal-3 >17.8 ng/mL were not factors associated with survival (see Model 4, Table 3).

**Table 2:** Univariate Cox proportional hazards analysis.

	HR^a^ [95% CI]	P
	(n = 202)	
Age, per 10 y	1.24 [0.92–1.67]	0.156
Gender, male = 1	0.51 [0.18–1.48]	0.224
Obesity	0.64 [0.25–1.66]	0.364
Diabetes	1.28 [0.57–2.85]	0.547
AHT	1.55 [0.75–3.17]	0.233
Dyslipidemia	0.90 [0.43–1.88]	0.786
ICM	1.42 [0.70–2.90]	0.331
Acute pulmononary *	1.48 [0.67–3.26]	0.342
LVEF per 5%	0.82 [0.69–0.98]	**0.029**
HR, per 5 unit	1.15 [1.04–1.26]	**0.005**
MRA	0.98 [0.48–1.98]	0.950
ARA II	0.19 [0.03–1.39]	**0.103**
Calcium blockers	0.94 [0.22–4.02]	0.940
AVK drugs	1.04 [0.49–2.21]	0.916
Antiplatelet Agents	1.03 [0.50–2.09]	0.943
Statines	0.71 [0.34–1.46]	0.355
Beta blockers	0.35 [0.15–0.79]	**0.002**
ACE inhibitors	0.51 [0.25–1.05]	**0.070**
Furosemide	2.78 [1.28–6.03]	**0.042**
Creatinine per 10 μM	1.11 [1.02–1.20]	**0.013**
Serum sodium per mM	0.82 [0.76–0.87]	**<0.001**
BNP per 100 pg/ml	1.09 [1.05–1.13]	**<0.001**
Gal-3 > 17.8 ng/ml	7.25 [3.28–16.03]	**<0.001**
Gal-3 per 10 ng/ml	2.45 [1.91–3.15]	<0.001

^a^ Hazard ratio adjusted for age and gender. In bold variables with p < 0.2 implemented in the multivariate cox proportional hazards analysis.

**Table 3:** Multivariate cox proportional hazards analysis.

	HR	95%CI	P
**Model 1**	4.75	1.99–11.36	**<0.001**
**Model 2**	7.01	3.01–16.30	**<0.001**
**Model 3**	6.07	2.62–14.02	**<0.001**
**Model 4**	7.25	3.28–16.03	**<0.001**
**Model 5**	5.75	1.97–16.81	**<0.001**

HR value for Gal-3 > 17.8 ng/mL adjusted for age, gender and in model 1 (+ creat, BNP, serum sodium) or in model 2 (+ ARA II, Beta blockers, ACE inhibitors, Furosemide) or in model 3 (+ LVEF and HR). Model 4, HR value for Gal-3 > 17.8 ng/mLadjusted for age, gender, MRA and interaction term MRA*Gal-3>17.8 ng/mL (0.87 (95% CI, 0.37–2.05), p = 0.750 and 0.57 (95% CI, 0.13–3.07). HR values for MRA and interaction term respectively). Model 5, HR values for Gal-3 > 17.8 ng/ml determined using stepwise method for selection of variables included in model 1 to 3.

## Discussion

Our study confirms previous observations on the prognostic value of Gal-3 in patients with HF [27]. Our cohort consisted of HF patients with systolic HF, but even in patients with preserved ejection fraction Gal-3 was shown to be prognostic [28]. Moreover, Gal-3 changes in patients were shown to be a strong prognostic indicator [29]. In addition, Gal-3 levels have a potential role in early detection of myocardial structural and functional alterations [30] and risk stratification in HF [31, 32].

Since patients with low Gal-3 levels were shown to benefit from statin therapy [33], this observation suggested that Gal-3 levels could be used to predict response to therapies. Therefore, we assessed the influence of MRA treatment on the prognostic value of Gal-3. Clearly, in our cohort MRA treatment did not alter the prognostic value of Gal-3. We also observed that MRA treatment did not alter mortality in our cohort. Several clinical studies have proposed that MRA treatment reduced all-cause death in HF [34–37] but other studies failed to show a reduction in mortality [23, 38]. However, the positive studies were conducted in very large cohorts and the beneficial effect of MRA may not be observed in the later rather small cohorts. Fiuzat et al. have also investigated the relationship between Gal-3 levels and the use of MRA in HF [23]. Despite the fact that this was a much larger cohort, the authors did not show a significant difference in outcomes of patients on MRA, even after adjustment for important clinical variables. Accordingly, as observed in our study, the prognostic value of Gal-3 in this study was not impaired by MRA use. We believe that a prospective randomized multicentric study will be required to further investigate the possible interactions of MRA and Gal-3, and to draw final conclusions whether Gal-3 can be useful to select patients who will benefit from MRAs.

### Study limitations

This study was retrospective and designed with the IBLOMAVED cohort. Despite the comparative groups were optimized with the use of the propensity score matching, the analysis was performed on a limited number of patients.

## Conclusions

In our cohort of chronic stable systolic HF, baseline Gal-3 has a strong independent prognostic value irrespective of treatment with MRAs. The significance of the observed lack of an interaction between Gal-3 and treatment effect of MRAs remains to be elucidated.
